# Diagnostics, Risk Factors, Treatment and Outcomes of Acute Kidney Injury in a New Paradigm

**DOI:** 10.3390/jcm9041104

**Published:** 2020-04-13

**Authors:** Charat Thongprayoon, Panupong Hansrivijit, Karthik Kovvuru, Swetha R. Kanduri, Aldo Torres-Ortiz, Prakrati Acharya, Maria L. Gonzalez-Suarez, Wisit Kaewput, Tarun Bathini, Wisit Cheungpasitporn

**Affiliations:** 1 Division of Nephrology, Department of Medicine, Mayo Clinic, Rochester, MN 55905, USA; charat.thongprayoon@gmail.com; 2 Department of Internal Medicine, University of Pittsburgh Medical Center Pinnacle, Harrisburg, PA 17105, USA; hansrivijitp@upmc.edu; 3 Division of Nephrology, Department of Medicine, University of Mississippi Medical Center, Jackson, MS 39216, USA; kkovvuru@umc.edu (K.K.); skanduri@umc.edu (S.R.K.); mgonzalezsuarez@umc.edu (M.L.G.-S.); 4 Department of Medicine, Ochsner Medical Center, New Orleans, LA 70121, USA; Aldo_t86@hotmail.com; 5 Division of Nephrology, Department of Medicine, Texas Tech University Health Sciences Center, El Paso, TX 79905, USA; prakrati.c.acharya@gmail.com; 6 Department of Military and Community Medicine, Phramongkutklao College of Medicine, Bangkok 10400, Thailand; wisitnephro@gmail.com; 7 Department of Internal Medicine, University of Arizona, Tucson, AZ 85724, USA; tarunjacobb@gmail.com

**Keywords:** acute kidney injury, acute renal failure, biomarkers, critical care, renal replacement therapy, risk factors outcomes, predictors

## Abstract

Acute kidney injury (AKI) is a common clinical condition among patients admitted in the hospitals. The condition is associated with both increased short-term and long-term mortality. With the development of a standardized definition for AKI and the acknowledgment of the impact of AKI on patient outcomes, there has been increased recognition of AKI. Two advances from past decades, the usage of computer decision support and the discovery of AKI biomarkers, have the ability to advance the diagnostic method to and further management of AKI. The increasingly widespread use of electronic health records across hospitals has substantially increased the amount of data available to investigators and has shown promise in advancing AKI research. In addition, progress in the finding and validation of different forms of biomarkers of AKI within diversified clinical environments and has provided information and insight on testing, etiology and further prognosis of AKI, leading to future of precision and personalized approach to AKI management. In this this article, we discussed the changing paradigms in AKI: From mechanisms to diagnostics, risk factors, and management of AKI.

## 1. Introduction

Acute kidney injury (AKI) is a highly complicated clinical disorder that is widely characterized by rapid rate of reduced rate of glomerular filtration (GFR), demonstrated by a rise in serum creatinine (SCr) concentration or oliguria, or both [1,2,3,4,5]. AKI is common among hospitalized patients, affecting approximately 10%–20% of hospitalized patients, of whom 10% require renal replacement therapy (RRT) [6,7,8,9,10,11]. Among critically ill patients, the incidence of AKI has been reported as high as 45–50% [2,12]. AKI is associated with significant morbidity, mortality, extra cost incurred in the hospitalization process, longer stay in the hospital, and long-term consequences, including chronic kidney disease (CKD) and end-stage kidney disease (ESKD) [13,14,15,16]. In the United States, AKI is associated with high hospitalization costs that range from $5.4 to $24.0 billion [17]. Overall mortality rate at 30 days post AKI is as high as 24% [18]. Each year, around 1.7 million people are globally thought to die from AKI [19].

In the recent years, there has been significant progress in the discovery and validation of AKI biomarkers in a number of clinical settings and has provided information and insight on diagnosis, prognosis as well as etiology of AKI [20]. Furthermore, the increasingly widespread use of electronic health records (EHR) across hospitals has substantially increased the amount of data available to investigators and has shown promise in advancing AKI research [21,22]. In this this article, we discussed the changing paradigms in AKI: from mechanisms to diagnostics, risk factors, and management of AKI.

## 2. Definition of AKI, Persistent AKI, and Renal Recovery after AKI

### 2.1. Definition of AKI

In 2012, the Kidney Disease Improving Global Outcomes (KDIGO) [23] gave out guidelines on the management of AKI to make the diagnosis process of the condition standardized and the severity of the disease based on absolute or relative increases in SCr and further progressive extent of oliguria, which built off of the RIFLE criteria [24] and the AKIN criteria [25], Table 1. KDIGO describe AKI as a condition that comprise of one or more of the following: (1) an increase in SCr level ≥0.3 mg/dL (≥26.5 µmol/L) within 48 h, or (2) an increase in SCr level to ≥1.5 times baseline, which is known or presumed to have occurred within the prior 7 days, or (3) a urine volume of less than <0.5 mL/kg/h for 6 h or longer.

### 2.2. Baseline SCr, Adjust SCr for Fluid Balance, and Body Weights for Urine Output Criterion

**Figure 1:**
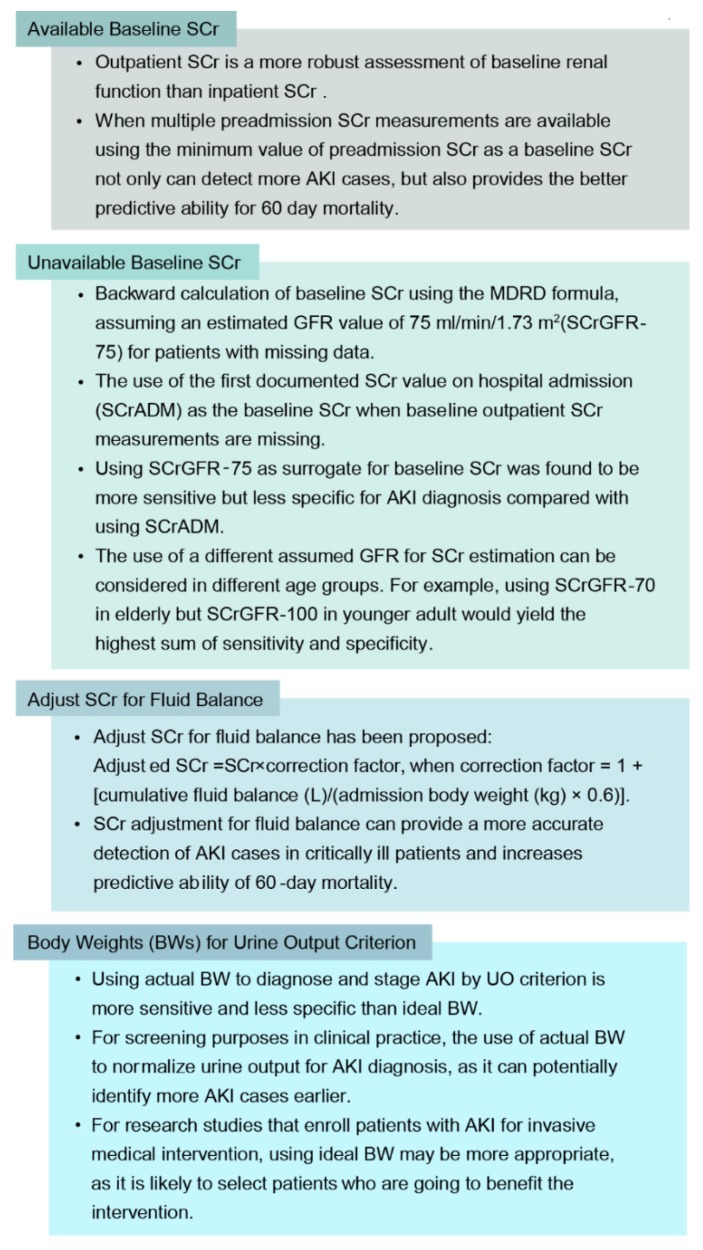
Baseline SCr, Adjust SCr for Fluid Balance, and Body Weights for Urine Output Criterion.

Establishing the baseline SCr is very much important in AKI diagnosis and classification [26,27]. Inaccurate determination of the baselines SCr can lead into misclassification of AKI and additionally impact the overall prognostication of the outcomes associated to AKI [28]. SCr at the outpatient is actually a very vigorous assessment of the renal function. The process is so robust such that it is more effective at the outpatient compared to the inpatients. This is mainly because it usually represents a kind of steady state and is not altered by the index critical condition of the illness [29]. When several preadmission SCr measurements are available, the use of minimum value of the preadmission SCr as the baseline SCr can detect more AKI cases, but also provides the better predictive ability for sixty day mortality [26] (Figure 1).

In clinical practice, it is very common that baseline outpatient SCr is unavailable [30]. While the Acute Dialysis Quality Initiative (ADQI) has recommended backward estimation of baseline SCr by applying the Modification of Diet in Renal Disease (MDRD) formula, making the assumption of an estimated GFR value of 75 mL/min/1.73 m^2^ (SCrGFR-75) for patients with no available baseline SCr [24], the European Renal Best Practice (ERBP) proposes the usage of the initial documented SCr value on hospital admission (SCrADM) as the baseline SCr when baseline outpatient SCr values are not available [31].

The types of strategies have their own shortcomings [24,31]. While backward calculation can lead to misclassification of AKI, especially in the early stages [32], the use of SCrADM as the baseline SCr can be inaccurate in patients who might be suffering from community-acquired AKI, as the SCr might had already escalated before the time of hospitalization [30,33]. In addition, using SCrGFR-75 as surrogate for baseline SCr was established to be more sensitive but less specific for AKI diagnosis compared with the use of SCrADM [30].

In clinical practice, prevention of AKI and subsequent timely treatment may improve the outcomes of the patient with AKI. Therefore, for the stratification risk purposes within the clinical undertakings, we highly encourage the application of SCrGFR-75 for the diagnosis of AKI, as it has the ability to properly identify more AKI cases. On the other hand, using SCrADM may be suitable for research studies, as it would be more likely to enroll patients who are going to benefit from the intervention [30]. Furthermore, since GFR decreases with age, the use of SCrGFR-75 might result in over-AKI classification in the elderly [34], therefore use of a different assumed GFR for SCr estimation could be considered in different age groups. For instance, applying SCrGFR-70 among the elderly and SCrGFR-100 for the younger adult would generate high amount of sensitivity together with specificity [30,34].

Among perioperative and intensive care unit (ICU) settings, volume overload is very common. It can cause the dilution and masking SCr increments, which may result in a delay in AKI diagnosis in critically ill patients [35]. Adjust SCr for fluid balance has been proposed with the following formula: adjusted SCr = SCr × correction factor, when correction factor = 1 + [cumulative fluid balance (L)/(admission body weight (kg) × 0.6)] [35,36,37]. SCr adjustment for fluid balance can provide a more accurate detection of AKI cases in critically ill patients and increases predictive ability of sixty day mortality [35].

Given the definition of AKI is currently based on absolute or relative changes in SCr and weight-adjusted hourly urine output [23], body weight (BW) is an essential factor and used when normalizing the UO for weight and time. Using actual BW to diagnose and stage AKI by UO criterion is more sensitive and less specific than ideal BW [38]. Thus, the choice of using ABW or IBW for AKI diagnosis and classification depends on the purpose of the AKI definition. In clinical practice, AKI prevention and early treatment of AKI may help improve patient outcomes. Therefore, for screening purposes in clinical practice, we suggest the use of ABW to normalize UO for AKI diagnosis, as it can potentially identify more patients with AKI earlier. Conversely, for research studies, using IBW may be more appropriate, as it is likely to select patients who are going to benefit the treatment [38].

### 2.3. Persistent AKI and Renal Recovery after AKI

Recently, the term “persistent AKI (pAKI)” has been proposed and is described as AKI diagnosis together with an increase in SCr that persisted through hospital discharge [39,40,41,42,43,44,45]. pAKI could be highly relevant endpoint for some additional further studies in the future [39]. As AKI which resolve very rapidly still has worse outcomes when compared to the patients who are not suffering from AKI, the overall outcome of transient AKI had been reported to be significantly better when comparison is made to pAKI [39,40,41,42,43,44,45]. pAKI is closely linked with more severe outcomes among patients when comparison is made to transient AKI, such as development of progressive CKD, increased mortality among those hospitalized, and reduced long-term survival [39,40,41,42,43,44,45].

The effects of renal recovery following AKI condition on the outcomes have recently been described [39,46,47], when complete renal recovery is defined as no AKI at patient discharge (comparing the SCr at discharge to the SCr at baseline). On the other hand, partial renal recovery is defined as AKI that is not complete renal recovery, and without the need for renal replacement therapy at discharge. No renal recovery is defined as a need for renal replacement therapy at discharge [39,46,47].

The Acute Disease Quality Initiative 16 Workgroup recently published a consensus report that placed more emphasis on the importance of renal recovery following AKI [46]. Recovery of renal function after AKI has been shown to be an independent determinant of morbidity and mortality in patients who are hospitalized, including those who are within ICU, or those who had undergone a process of cardiac surgery [40,48,49,50].

## 3. Causes and Diagnosis of AKI

**Figure 2:**
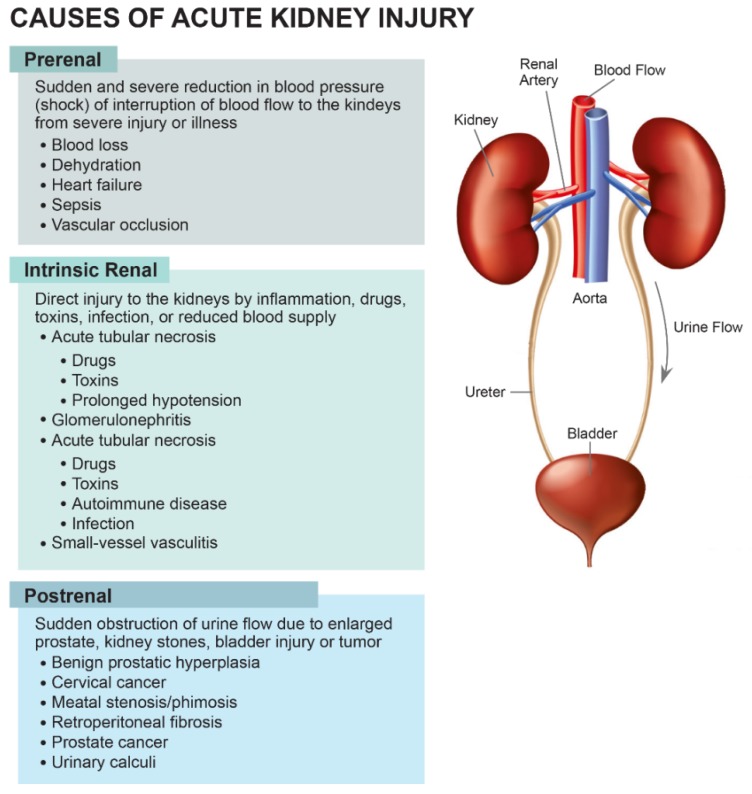
Causes of AKI.

The main causes of AKI are divided into three categories: Prerenal, intrinsic renal and postrenal (Figure 2).

AKI can have many different causes as shown in Figure 2, such as decreased kidney perfusion, parenchymal kidney diseases, acute tubular necrosis (ATN), and obstruction of the urinary tract. Articles on detailed specific causes of AKI have been published in our current special issue “Diagnostics, Risk Factors, Treatment and Outcomes of Acute Kidney Injury in a New Paradigm” (https://www.mdpi.com/journal/jcm/special_issues/acute_kidney_injury) [3,51,52,53,54,55,56,57,58,59,60,61,62,63,64,65,66,67,68,69,70,71,72,73,74,75,76,77,78,79,80,81,82,83,84,85,86,87,88]. Reported incidence of AKI is different among different patient populations as shown in Table 2.

In patients with AKI from some other causes, urinalysis, dipstick, sediment, albuminuria and total proteinuria; and the presence or absence of hematuria, pyuria, renal tubular epithelial cells, and granular and cellular casts, chemistries, and serologic evaluation can be helpful in identifying the cause of AKI, as shown in Table 3. Imaging studies are usually performed to evaluate the presence of hydronephrosis, defined as dilatation of the renal collecting system due to obstruction [1].

Furosemide “stress test” (administration of 1 mg/kg of IV furosemide with 1:1 replacement of urine output with saline) can be used to assess prognosis: Patients with <200 mL of urine output over the subsequent 2 h are at greater risk for progression to a higher AKI stage or to the need for RRT [107,108].

## 4. Biomarkers of Acute Kidney Injury (AKI)

SCr level does not detect AKI promptly and increased SCr and oliguria may not occur for several hours after the onset of an acute decline in GFR [1]. In addition, the rise in SCr (and decrease in estimated GFR) may be delayed in patients with low muscle mass or volume overload and faster in those with high muscle mass or volume depletion [1,109].

Within the last few years, the discovery and further validation of the special biomarkers of the kidney injury has attracted great attention [110]. Several biomarkers like Cystatin C and further neutrophil gelatinase-associated lipocalin have consequently been recommended for the purpose of diagnosis, severity grouping and more essential, the modification in the AKI outcome [1]. Novel biomarkers are under investigation to determine whether they may enable earlier detection of decreased GFR and complications of AKI [3,111], as shown in Table 4.

## 5. Risk Factors

While diabetics with baseline CKD represent the highest risk patient population for AKI development [128], overall reported risk factors for AKI from the literature include older age, history of diabetes, hypertension, congestive heart failure, peripheral vascular disease, sepsis, use of nephrotoxic drugs, higher severity of disease scores, use of vasopressors/inotropes, high risk surgery, emergency surgery, hemodynamic instability, use of intra-aortic balloon pump, anemia requiring blood transfusion, and longer time in cardiopulmonary bypass pump [129,130,131,132], Table 5.

## 6. Outcomes and Mortality Risk among Patients with AKI

AKI is associated with significant morbidity and mortality [13,14,15]. Patients with AKI who fail to recover their renal function, have been reported to be having 47% hospital mortality. In addition, among those who are discharged from the hospital alive, 1-year patient survival is only 77% [47]. Mortality risk among patients with AKI in different patient populations are demonstrated in Table 6. In addition to increased mortality, AKI is also associated with an increased risk of cardiovascular mortality and major cardiovascular events, particularly heart failure and acute myocardial infarction [133].

Irrespective of cause, the severity of AKI is related to the risk for complications [1]. Complications of AKI result from impaired excretory, endocrine, and metabolic kidney functions. Decreased GFR and tubular function lead to retained water and solutes, manifested by volume overload, hyperkalemia, high an- ion gap metabolic acidosis, hyponatremia, hyperphosphatemia, hypermagnesemia, encephalopathy, pericarditis, pruritus, and bleeding due to platelet dysfunction [1]. Drug toxicity is common because of altered pharmacokinetics and pharmacodynamics. Complications may occur in other organ systems throughout the course of disease; multiple organ failure is associated with the highest mortality. Such form of injuries are mainly recorded in close to 20% of the patients who have been hospitalized, with the great complications recorded to comprise of drug toxicity, uremic complications, disorders of the electrolyte and subsequently volume overload. Incomplete recovery may lead to new onset or worsening of CKD [1].

## 7. AKI Prevention and Management of AKI

### 7.1. AKI Prevention

Since severe AKI is associated with a high mortality rate and there are currently no effective targeted pharmacotherapies available for AKI, all the relevant measures that are undertaken for the purpose of preventing AKI (Table 7).

Fluid composition has also been the subject of substantial investigation. The use of hydroxyethyl starch has been shown to result in increased rates of AKI especially in patients with sepsis [151,152], while on the other hand, saline has been demonstrated to increase the risks associated with dialysis, mortality and continuous renal dysfunction when compared to the fluids that are very similar to the relevant physiological ones like the Ringer’s lactate solution [153,154].

General measures undertaken to limit the risk comprise of the prevention as well as the treatment of volume depletion and avoidance of nephrotoxic drugs [155]. IV isotonic fluids before, during, and after intra-arterial administration of iodinated radiocontrast media may reduce risk for contrast-induced AKI [98,156,157,158]. Monitoring therapeutic levels of nephrotoxic drugs, such as vancomycin, aminoglycosides, and calcineurin inhibitors, can reduce risk for AKI. KDIGO suggests additional measures to reduce the risk for nephrotoxicity of aminoglycosides and amphotericin B [1].

While the data on discontinuation of the continued angiotensin-converting enzyme inhibitors (ACEIs) as well as the angiotensin-receptor blockers (ARBs) during the period of acute illness to prevent AKI is controversial [159,160], some other nephrotoxic drugs need to be avoided especially among people who are suffering from CKD, such as nonsteroidal anti-inflammatory drugs (NSAIDs) [161]. Contrast-associated AKI is becoming less frequent because of reduced toxicity and lesser amounts of contrast media used for imaging techniques. However, prevention measures should still be considered for individual patients, especially in patients with CKD [2].

### 7.2. Management of AKI

The timely identification of the at risk patents, timely diagnosis and early treatment of all the AKI cases are essential part of the general management of individual patients who might be suffering from AKI. The initial principle of AKI management is specifically to treat its causative factor or trigger, such as treating infection in sepsis-associated AKI. The second principle of management and specific treatments according to the underlying cause of AKI syndromes such as hepatorenal syndrome, cardiorenal syndrome, glomerulonephritis, interstitial nephritis, vasculitis, and multiple myeloma, etc.. Currently, there are currently no effective pharmacotherapies for treating ATN. The specific treatments for these specific types of kidney injuries are not focus of this review. The third principle is based on ensuring that there is avoidance of any additional insults of AKI. There is need to optimize hemodynamics that are systemic based, so that even in the absence of some other triggers, additional damage is not experienced and correct perfusion pressure and renal perfusion are adequately maintained. The fourth principle is to apply provide supportive care to prevent and treat complications.

RRT are a spectrum of dialysis modalities employed in management of renal dysfunction. They are broadly classified as continuous, intermittent and hybrid variants. Continuous renal replacement therapies (CRRT) are ideally used in hemodynamically unstable patients to allow steady solute and volume shifts. CRRT further categorized based on principles of clearances [162] to four types. Slow continuous ultra-filtration (SCUF) aims at filtration of plasma water in patients with refractory volume overload while no significant solute clearances are achieved. The other three modalities are continuous veno-venous hemofiltration (CVVH) (convection), continuous veno-venous hemodialysis (CVVHD) (diffusion) and continuous veno-venous hemodiafiltration (CVVHDF) (diffusion and convection) [163,164,165,166]. Peritoneal dialysis, a slow efficient continuous modality is an acceptable alternative to extra corporeal modalities [167]. Intermittent hemodialysis (iHD) is routinely prescribed for 3 to 4 h three times a week and supports rapid clearances of small molecules and toxic drugs [168]. Hybrid therapies include sustained low-efficiency dialysis (SLED), prolonged intermittent renal replacement therapy (PIRRT), extended daily dialysis with filtration (EDDf) and accelerated veno-venous hemofiltration (AVVH) [169]. Hybrid modalities tend to blend features of intermittent and continuous modalities with objectives to enhance hemodynamic stability while minimizing the disadvantages of continues modalities [170].

Even though CRRT has multiple potential advantages, no randomized control studies (RCT) have proven survival benefit of any specific modality [171,172]. In a metanalysis by Nash et al. including 21 studies comparing iHD, SLED and CRRT modalities failed to demonstrate mortality difference or superior renal recovery of one over other groups [173]. Friedrich et al. performed a systematic review involving 19 RCTS, compared outcomes among hemofiltration and hemodialysis in patients with AKI and were unsuccessful in achieving survival advantages of one therapy over other. However, hemofiltration group had a trend towards increased clearance of inflammatory molecules including cytokines [174].

Currently, recommended effluent CRRT dose for clinical practices is 20 to 25 mL/kg/h. Multiple studies were conducted comparing different doses. The prospective randomized study by Ronco et al. involving critically ill AKI patients comparing three different doses (20 mL/kg/h, 35 mL/kg/h and 45 mL/kg/h) reported inferior survival rates in in lower dose group (20 mL/kg/h) compared to higher (35 mL/kg/h and 45 mL/kg/h) [175]. However, the land mark RCTs, VA/NIH Acute Renal Failure Trial Network (ATN trial) [176] by Palevsky et al. (35 mL/kg/h vs. 20 mL/kg/h) and RENAL study by Bellamo et al. (40 mL/kg/h vs. 25 mL/kg/h) [177] were ineffective in decreasing mortality, or improving renal recovery at higher dose compared to lower. In a metanalysis by Clark et al, including 4 RCTs comparing high volume hemofiltration (>50 mL/kg/h) (HVHF) to standard volume hemofiltration (SVHF) among septic AKI patients did not demonstrate any difference in 28-day mortality. Even though vasopressor requirements were lower among HVHF group, they sustained significant adverse effects including hypokalemia, hypophosphatemia, metabolic alkalosis, excessive micronutrient loss [178].

Significant controversies exist regarding the timing of initiation of RRT and are still a topic of debate. HEROICS study, a prospective randomized multicenter trial including post-cardiac surgery septic shock patients compared early HVHF (80 mL/kg/h) to delayed CVVHDF with no difference in outcomes (30 day mortality) [179]. The Artificial Kidney Initiation In Kidney Injury (AKIKI) trial and Initiation of Dialysis Early Versus Delayed in the Intensive Care Unit (IDEAL- ICU) trials are randomized multicenter studies involving critically ill AKI patients with KDIGO stage3 compared early (Immediately after randomization or within 12 h) vs. delayed initiation of RRT, (refractory to medical management or >48 h) were unsuccessful in demonstrating outcome benefits in early group as compared to delayed [180,181]. The Effect of Early vs. Delayed Initiation of Renal Replacement Therapy on Mortality in Critically Ill Patients with Acute Kidney Injury (ELAIN) study did report a 15% reduction in 90-day mortality in early initiation group, however this study suffered serious criticism including single centered study with significant number of post cardiac surgical patients, RRT initiation at KDIGO stage 2 and less than 24 h difference in initiation of RRT among both groups [182].The current evidence suggests no tremendous benefit of early initiation of RRT but is associated with complications. Therefore, the timing of initiation of RRT should be individualized based on judicious examination, disease acuity and medical necessity [183]. The two large ongoing multicenter RCTS, AKIKI-2 [184] and START- AKI [185] trials might further shed some light on Ideal timing of RRT.

## 8. Potential Directions and Future Scope

**Figure 3:**
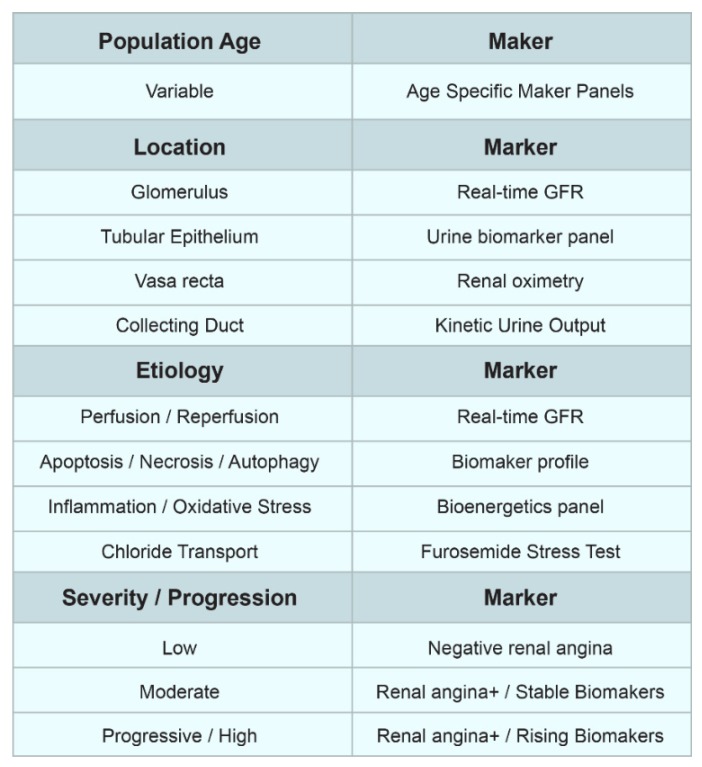
Future of biomarkers of AKI. Abbreviations: GFR, glomerular filtration rate.

Two advances from past decades, the usage of the computer decision intelligence and the discovery of AKI biomarkers, have the great ability to generally improve the approach applied in diagnosis and further treatment of AKI. For instance, in the instance of AKI, electronic, the automated diagnostic strategy tend to create great opportunity to initiate predictive strategies, subsequently optimize the relevant AKI alerts, and subsequently trace AKI events across various associations, as well as the relevant managerial datasets [186]. In addition, dynamic and multidimensional approach to AKI, using AKI biomarkers over time, will be presented as a versatile theoretic construct usable to characterize and phenotype AKI itself, refining the precision of diagnosis and making possible the ability to track different aspects of the injury as they change over time, potentially leading to a modern and personalized approach to AKI [187] (Figure 3).

## 9. Conclusions

Based on the mere fact that presently there is absence of effective pharmacotherapies that are usable for AKI, all measures geared toward preventing the condition should be taken seriously. Two advances from past decades, the usage of computer decision intelligence support and the discovery of AKI biomarkers, have the great ability to in a sustainable way improve the general diagnostic strategy to AKI and its further treatment. The advances in developments and future progress in AKI biomarkers over time can lead to future of precision and personalized approach to AKI management.

## Figures and Tables

**Table 1:** KDIGO criterion for diagnosis and staging of AKI [23].

Stage	Serum Creatinine	Urine Output
1	1.5–1.9 times baseline OR 0.3 mg/dL increase	<0.5 mL/kg/h for 6–12 h
2	2.0–2.9 times baseline	<0.5 mL/kg/h for ≥12 h
3	3.0 times baseline OR Increase in serum creatinine to ≥4.0 mg/dL OR initiation of replacement therapy	<0.3 mL/kg/h for ≥24 h OR anuria for ≥12 h

**Table 2:** Reported incidence of AKI is different among different patient populations [6,7,8,9,10,11,59,65,70,72,89,90,91,92,93,94,95,96,97,98,99,100,101,102,103,104].

Patient Populations/Settings	Incidence of AKI
- General hospitalized patients - ICU - Cardiac surgery - Transcatheter Aortic Valve Replacement - Sepsis - Acute respiratory distress syndrome - Extracorporeal Membrane Oxygenation ○ AKI ○ Severe AKI requiring RRT - Liver transplantation ○ AKI ○ Severe AKI requiring RRT	10%–20% 20%–50% 30%–50% 28% 16%–25% 44%–50% 63% 45% 41% 8%
- Lung transplantation ○ AKI ○ Severe AKI requiring RRT - Cardiac Transplantation ○ AKI ○ Severe AKI requiring RRT - Hematopoietic Stem Cell Transplantation ○ AKI ○ Severe AKI requiring RRT - Total Hip Arthroplasty ○ AKI - Severe AKI requiring RRT	53% 9% 47% 12% 55% 8% 6% 0.5%

AKI, acute kidney injury; ICU, intensive care unit; RRT, renal replacement therapy.

**Table 3:** Diagnostic tests in patients with AKI [1,105,106].

Diagnostic Test	Findings	Pathologic Condition (s)
Urinalysis with microscopy	Hyaline cast	Prerenal azotemia
	Muddy brown cast	ATN
	Dysmorphic RBC & RBC casts	GN
	WBC casts	AIN
	Crystals	Crystal induced nephropathy, drugs, nephrolithiasis
	Monomorphic RBCs, WBCs	UTI, Nephrolithiasis, Genitourinary tumors etc
	Protein	GN, Monoclonal gammopathy
CBC with peripheral smear	Anemia, Schistocytes, low platelets	TMA
Serum osmolality	Osmolar gap & severe metabolic acidosis	Toxin
Creatinine kinase	>5000 IU/L	Rhabdomyolysis
Serologic tests	HIV antibody	HIV associated nephropathy, HIV induced immunocomplex kidney disease
	Hepatitis serology	Membranous GN, MPGN
	ANA, dsDNA	Lupus nephritis
	C- ANCA, P- ANCA	ANCA vasculitis
	Rheumatoid factor, Cryoglobulins	Cryoglobulinemia, MPGN
	Anti—GBM antibody	Good pasture syndrome
	ASO	Infection related GN
	Low Complement levels	Lupus, Infection related GN, atheroemboli, MPGN, shunt nephritis
Fractional excretion of sodium (FeNa) *	<1%	Prerenal azotemia
Fractional Excretion of urea (Fe Urea)	<35%	Prerenal azotemia
POCUS (Volume Assessment)	IVC diameter ↓ (>50% w/inspiration)	Hypovolemia
Renal USG	Hydronephrosis, Hydroureter	Nephrolithiasis, Retroperitoneal fibrosis, BPH, Phimosis, Ureteral obstruction
	Renal vein thrombosis	Hypercoagulable state
Renal biopsy	Variable	GN, ATN, AIN, crystal induced nephropathy
Newer biomarkers	↑ NGAL, KIM 1, (TIMP-2)∙(IGFBP7) **	"Damage biomarkers" increased much before rise in creatinine

ATN: Acute tubular necrosis, GN- Glomerulonephritis, AIN: Acute interstitial nephritis, UTI: Urinary tract infection, ANA: Antinuclear antibody, ANCA: Antinuclear cytoplasmic antibody, GBM: Glomerular basement membrane, MPGN: Membranoproliferative glomerulonephritis, ASO: Anti Streptolysin, POCUS: Point of care ultrasound, IVC: Inferior vena cava, NGAL: neutrophil gelatinase–associated lipocalin, KIM-1: Kidney injury molecule -1, TIMP 2- Tissue inhibitor of metalloproteinases-2, IGFBP7: Insulin like growth factor-binding protein 7. Notes: UA dipstick ++ for blood but no RBCs - Suspect rhabdomyolysis. If urine protein creatinine ratio quite elevated but urine dipstick with low grade proteinuria - Suspect multiple myeloma. BUN out of proportion to Cr - Suspect GI bleeding, high dose steroids, high protein feeding. Urine eosinophils have low sensitivity (30.8%) and specificity (68.2%) for AIN^1^ so not diagnostic of AIN. * FeNa is affected in CKD, diuretics, contrast administration, acute GN and Rhabdomyolysis so is not quite reliable in cause of AKI diagnosis. ** FDA approved in 2014.

**Table 4:** Characteristics of selected novel biomarkers for acute kidney injury [112,113,114,115,116,117,118,119,120,121,122,123,124,125,126,127].

Novel Biomarkers	Specimen	Type	Representation	Study Population
NGAL	Serum, urine	Upregulated protein	Distal tubules	Cardiac surgery, Critically ill, CRS, KT
KIM-1	Urine	Upregulated protein	Proximal tubules	Cardiac surgery, KT
L-FABP	Urine	Upregulated protein	Proximal tubules	Cardiac surgery, Critically ill
IL-10	Urine	Cytokine	Inflammatory cascades	Cardiac surgery
IL-18	Urine	Cytokine	Inflammatory cascades	Cardiac surgery, Critically ill, KT
Urine Cystatin C	Serum, urine	Functional	Proximal tubules (urine), glomerular (serum)	Critically ill
NAG	Urine	Enzyme	Proximal tubules	Critically ill, KT
IGFBP7	Urine	Upregulated protein	Proximal tubules	Critically ill, cardiac surgery
TIMP-2	Urine	Upregulated protein	Proximal tubules	Critically ill, cardiac surgery
Calprotectin	Urine	Upregulated protein	Renal inflammation	Hospitalized patients
AGT	Urine	Enzyme	Renin-angiotensin activation	Heart failure
microRNA	Urine	RNA fragment	Proximal and distal tubules	Cardiac surgery

AGT, angiotensinogen; CRS, cardiorenal syndrome; IGFBP, insulin-like growth factor-bind protein 7; IL, interleukin; KIM-1, Kidney injury molecule-1; KT, kidney transplantation; L-FABP, liver fatty acid; LMWP, low-molecular weight protein; NAG, N-acetyl-b-D-glucosaminidase; NGAL, neutrophil gelatinase-associated lipocalin; TIMP-2, tissue inhibitor of metalloproteinase 2.

**Table 5:** Risk factors for AKI [129,130,131,132].

Modifiable	Non-Modifiable
Anemia/Blood transfusion Hypertension Hypercholesterolemia Hypoalbuminemia Infection/Sepsis Mechanical ventilator Nephrotoxic agents Use of vasopressors/inotropes High risk surgery Emergency surgery Hemodynamic instability Use of intra-aortic balloon pump Longer time in cardiopulmonary bypass pump	Chronic kidney disease Chronic liver disease Congestive heart failure Diabetes mellitus Older age Peripheral vascular disease

**Table 6:** Mortality outcomes of acute kidney injury in different patients’ population from selected studies [59,70,90,92,134,135,136,137,138,139,140,141,142,143,144,145,146,147,148,149,150].

Patient Populations	Odds Ratio (95% CI) for Mortality
Acute coronary syndrome	4.1 (3.3–5.0)
Cardiac surgery	6.27 (3.6–11.0)
TAVR	18.0 (6.3–52.0)
ECMO	3.7 (2.9–4.9)
Liver transplantation	3.0 (2.3–3.8)
Cirrhosis	2.6 (1.5–4.7)
Lung transplantation	1.5 (1.1–1.9)
Stem cell transplantation	3.0 (2.1–4.5)
Heart transplantation	2.7 (1.6–3.3)
Critically ill patients	1.4 to 2.5
Rhabdomyolysis	3.3 (1.1–9.7)
Cardiorenal syndrome	4.9 (3.7–6.5)
Burn patients	11.3 (7.3–17.4)
Ischemic stroke	2.5 (1.5–4.1)
Cancer	3.0 (2.3–3.9)
COPD	1.8 (1.6–2.0)
Malnutrition	2.0 (1.5–2.7)
Gastrointestinal bleeding	2.6 to 4.9

COPD, chronic obstructive pulmonary disease; ECMO, extra-corporal membrane oxygenation; NSAID, non-steroidal anti-inflammatory disease; TAVR, transcatheter aortic valve.

**Table 7:** AKI prevention measures.

**General Measures**	
**Identify patients at risk**	- Personal risks: older age, history of CKD, diabetes, dementia, coronary artery disease. - Related to clinical scenario: reason for admission, severity of illness, ICU stay, and recurrent hospitalizations.
**Use of Clinical decision support systems (CDSS)**	- Electronic-based alert systems in the hospitals have shown to improve the detection of AKI.
**Maintain euvolemia**	- Use intravenous fluids if hypovolemia is anticipated in clinical settings such as poor oral intake, vomiting, diarrhea, polyuria, etc. - Avoid starches for volume resuscitation - Avoid volume overload by discontinuing fluids when appropriate.
**Avoid nephrotoxic medications.**	- Discontinue medications such as NSAIDs - Avoid ACE/ARB inhibitors (controversial) which affect the hemodynamics of the kidneys. - Avoid nephrotoxic antibiotics such as aminoglycosides, amphotericin and vancomycin. If their use is necessary, monitor levels if appropriate. - Utilize minimal dose and for the shortest time possible.
**Judicious use of contrasted studies**	- Outweigh risks vs. benefits of contrasted studies. Intra-arterial pose a higher risk than intravenous contrasted studies.
**Avoid hypotension**	- Decrease in renal blood flow is a known risk factor for AKI. It is therefore imperative to keep MAP >65 (target 65–70 mmHg), and a higher target (80–85 mmHg) in chronically hypertensive patients. - If vasopressors are too be used in the ICU, norepinephrine should be the first-choice to protect kidney function.
**Renal function monitoring**	- Monitor SCr as often as necessary, depending on the risk factors and clinical scenario. - Monitor fluid input and urinary output.
**Specific Clinical Scenarios**	
**Patients undergoing a procedure needing IV contrast use**	- Discontinue nephrotoxic medications - IV hydration with intravenous isotonic saline at a rate of 1 to 1.5 mL per kilogram per hour for 12 h before and up to 24 h after the procedure. A shorter protocol for patients undergoing urgent procedures comprises an intravenous infusion of isotonic saline for 1 to 3 h before and 6 h after the procedure. - Recent data does not support the use of IV bicarbonate or N-acetyl cysteine. - Utilize low-osmolar or iso-osmolar contrast media. - Minimize contrast volume (<350 mL or <4 mL per kilogram)
**Traumatic and non-traumatic rhabdomyolysis**	- Early and aggressive volume expansion with isotonic solutions aimed at increasing urine flow (about 200–300 mL/h). - Use of bicarbonate is not evidence based and might precipitate metastatic tissue calcification and ionized hypocalcemia - Use of diuretics is not generally recommended.
**Patients undergoing cardiac surgery**	Preoperative: - Perform pre-operative AKI stratification. - Delay elective surgeries if current AKI and delay 24–72 h after contrast use. - Discontinue ACE/ARB (controversial) - Discontinue NSAIDs. - Limited use of blood transfusions. - Correcting hypoalbuminemia with exogenous albumin preoperatively may play a role in preventing AKI. - Use of balanced crystalloid solutions guided by measures of fluid responsiveness. Intraoperative - Cold perfusion of the kidneys during aortic aneurysm repair - Avoidance of hyperthermia. - Pulsatile Cardiopulmonary bypass. - Avoidance of hemodilution. - Use of volatile anesthetics. - Minimization of aortic manipulation. - Techniques to prevent procedure-related atheroembolism. Postoperative - Low tidal volume strategy. - General measures mentioned above. - Glucose control (target 127–179 mg/dL).

AKI; acute kidney injury; CKD, chronic kidney disease; ICU, intensive care unit; NSAIDs, nonsteroidal anti-inflammatory drugs; SCr, serum creatinine; ACE, angiotensin converting enzyme; ARB, angiotensin-receptor blocker; MAP, mean arterial pressure; IV, intravenous.
